# Fluid Balance and Thermoregulatory Responses during Wheelchair Basketball Games in Hot vs. Temperate Conditions

**DOI:** 10.3390/nu14142930

**Published:** 2022-07-17

**Authors:** Fabian Grossmann, Claudio Perret, Bart Roelands, Romain Meeusen, Joelle Leonie Flueck

**Affiliations:** 1 Swiss Paraplegic Centre, Sports Medicine, 6207 Nottwil, Switzerland; claudio.perret@paraplegie.ch (C.P.); joelle.flueck@sportmedizin-nottwil.ch (J.L.F.); 2 Human Physiology and Sports Physiotherapy Research Group, Vrije Universiteit Brussel, 1050 Brussels, Belgium; bart.roelands@vub.be (B.R.); romain.meeusen@vub.be (R.M.)

**Keywords:** sports nutrition, paralympics, fluid intake, sweating

## Abstract

The impaired vaso- and sudomotor functions limit sweat capacity in individuals with a spinal cord injury (SCI) and might increase the risk for heat-related illness and decreased performance, especially in hot conditions (HOT). This study investigated the differences in fluid balance and thermal responses between wheelchair basketball (WCB) games in HOT and temperate conditions (TMP). Eleven male WCB athletes (39.8 y, 82.8 kg) with SCI (lesion level C5-L4) participated, five in HOT (31 °C) and eight in TMP games (21 °C). Fluid balance, sweat rate, body core temperature, distance, velocity and thermal sensation were assessed. The relative change in body mass was higher in the HOT group (median: −0.35%, interquartile-range: 0.15%, *p* = 0.02) compared to TMP (+0.11%, 0.35%) group. The sweat rate was significantly higher in the HOT group (0.93 L/h, 0.58 L/h, *p* = 0.02) compared to the TMP groups (0.48 L/h, 0.19 L/h). Body core temperature increased significantly higher in the TMP group (1.05 °C, 0.15 °C, *p* = 0.01) compared to the HOT group (0.8 °C, 0.4 °C). The mean velocity (HOT: 1.12 m/s, 0.11 m/s, TMP: 1.07 m/s, 0.08 m/s, *p* = 0.54) did not differ between the games. The WCB game in HOT leads to significantly higher sweat rate and loss in body mass compared to TMP. Even relative body mass loss was less than 2%. Athletes thus have to be supported with enough fluid, especially during games in HOT.

## 1. Introduction

In individuals with a spinal cord injury (SCI), sensorimotor and autonomic functions are impaired [1]. Due to damage to the autonomic nervous system, cardiovascular, respiratory, bladder, bowel, sexual and thermoregulatory functions can be negatively affected [1]. A more detailed introduction of the injury would be beyond the scope of this article, but an excellent summary of the consequences of SCI can be found in the work of Hou and Rabchevsky [1]. As a consequence, not only daily life but also athletic performance can be influenced in an adverse way [2]. The afferent input to the thermoregulatory center below the lesion level is decreased, which leads to reductions in efferent output, causing an impairment of the cutaneous vasodilatation as well as decreased activation of the sweat glands [3,4,5,6].

During exercise, energy metabolism is increased, and the main part of this energy is released as heat. To regulate body core temperature (Tc), it is essential that the produced heat can be dissipated rapidly [7]. The primary thermoregulatory reflexes to dissipate heat are: an increase in sweating for evaporative heat loss and an increase in skin blood flow for heat loss by convection and radiation [7,8]. Thus, the higher the lesion level, the larger the risk for overheating and consequently for reduced performance or heat-related illnesses (e.g., dizziness, heatstroke) in athletes with SCI [5]. In hot environmental conditions, this risk is accentuated.

In able-bodied (AB) athletes, sweat rate (SR) is dependent on different factors such as exercise intensity, fitness level, sex, acclimatization and environmental conditions (i.e., hot temperatures, high relative humidity) [9,10] and varies from 0.3 to 2.4 L/h [11]. In team sports, physiological demands of exercise (e.g., level of physical performance, technical skills) vary widely depending on the level of competition (i.e., lowest national league vs. Olympic Games), the field/court position of the player and the characteristics of the game [10]. Additionally, SR shows a high inter-individual variation. Compared to outdoor activities, the SR of indoor sports (e.g., basketball, volleyball) can be increased since, at standstill, the airflow around the skin is smaller, and therefore cooling is reduced [10]. Inadequate hydration strategies can lead to hypohydration [10]. Significant hypohydration, such as the loss of more than 3 to 5% of body mass, impairs cognition and technical and physical performance [11,12]. A greater loss of body mass (6–10%) impairs exercise performance (i.e., decrease in cardiac output, sweat production and skin- and muscle blood flow) to a higher extent. Therefore, in AB athletes, fluid replacement strategies are greatly appreciated in order to prevent any impairment of performance and reduce the risk of heat stress [13]. Individual variability in drinking behavior has been found in AB, which resulted in differences in hydration status ranging from substantial dehydration to hyperhydration [11]. Observed fluid intake differed from sport to sport (e.g., 0.3–0.5 L/h in soccer up to 1.6 L/h in basketball) [10,12]. As a rough recommendation, athletes might ingest 0.4 to 0.8 L/h of fluids or show a loss of body mass of less than 2% [11]. Different factors such as drinking opportunity, fluid palatability, the fear of weight gain during exercise or gastrointestinal problems influence the total fluid intake [10]. Furthermore, it has to be considered, that fluid absorption is influenced by osmolarity, the electrolyte and carbohydrate content of the fluid, which may affect total fluid intake [14,15]

The lesion of the spinal cord also affects the autonomic nervous system which is—among other functions—responsible for the regulation and secretion of glands [16]. Therefore, in athletes with SCI, the level of lesion, as well as the completeness of the injury, determines the number of active sweat glands and in such a way that the amount of potential sweat loss besides individual variations in SR [17]. In temperate conditions, it is well-known that individuals with tetraplegia (TP) present the lowest SR compared to those with high-level paraplegia (PA), low-level PA and AB [5]. Only a small amount of data are available for wheelchair team sports, since most studies included not only athletes with SCI but also individuals with, for example, an amputation. In wheelchair rugby, fluid loss was lower in SCI compared to AB, whereas both groups drank the same amount of fluid [18]. In wheelchair basketball (WCB), Logan-Sprenger and Mc Naughton [19] found an average sweat loss of one liter during the game. The fluid that athletes had voluntarily consumed replaced ~69% of sweat loss, which resulted in a mean body mass loss of 0.4% for SCI and 0.6% for non-SCI WCB players.

Fluid balance in hot conditions was investigated in a few studies [20,21]. Goosey-Tolfrey, Diaper, Crosland and Tolfrey [20] found that the amount of water ingested by wheelchair tennis players (~1198 mL) was nearly equivalent to the total sweat loss (~1300 mL) during a tennis session in the heat (30.4 °C). Price and Campbell [21] observed a larger fluid loss during wheelchair ergometry in individuals with high- and low-level PA (~0.7 L/h), compared to those with TP (~0.3 L/h). Interestingly, individuals with TP consumed the highest amount of fluid (~0.7 L/h). By reason of a low SR, drinking resulted in a gain in total body mass (~0.4 kg). The authors concluded that the overconsumption of fluid was an attempt to attenuate perceived heat stress [21]. Both low- and high-level PA showed a negative fluid balance (~−0.4 kg). In WCB, players mostly have a PA or TP with an incomplete transection of the spinal cord and therefore, cooling through increasing the SR should be partially functioning. Therefore, when individualized hydration strategies are applied in PA or incomplete TP individuals, any negative effect on performance due to hypohydration or overhydration in complete TP would not be anticipated. This is in line with the results from two studies, investigating SR and fluid intake during competitive games [18,19]. However, to our knowledge, no study compared fluid balance or thermoregulatory markers in individuals with SCI during games in different environmental conditions (i.e., hot versus temperate). Therefore, the aims of the present study were to describe and compare fluid intake, fluid loss, SR and thermoregulatory markers (i.e., Tc and perceived thermal sensation (ThS)) during competitive WCB in hot and temperate environmental conditions. Additionally, mean velocity and total distance covered were assessed to determine performance.

## 2. Materials and Methods

### 2.1. Participants

In total, eleven male WCB players volunteered to participate in the present investigation (Table 1). Inclusion criteria were age between 18 and 60 years, male, SCI, valid classification for WCB and member of a national team or of a team playing in the European League. Exclusion criteria were the inability to swallow the core temperature pill, other reasons for paralysis than a traumatic SCI (i.e., spina bifida, multiple sclerosis) and chronic diseases which affect body metabolism (i.e., hyper-/hypothyroidism). The included athletes were all tattoo-free. All participants were informed about the experimental protocol, both orally and written, and all participants provided their written, informed consent prior to data collection. The study was approved by the local ethical committee EKNZ (Approval-Nr.: 2018-01792, Basel, Switzerland) and registered on clinicaltrials.gov (accessed on 19 October 2018) (ID NCT03815708). All procedures were conducted according to the Helsinki Declaration [22].

### 2.2. Procedure

Data were assessed during two competitive WCB games (European League tournament), played over four quarters of 10 min (effective time) on two separate days. The sports hall was naturally heated, and the environmental condition was 30.3 °C with 52% relative humidity (rh) in game one (HOT). The natural environmental condition in Game two (TMP) was 21.6 °C with 30% rh. Since the HOT game was at the end of the season and TMP game at the beginning of the new season, several players changed the teams or retired. Therefore, the games were played by different players. Participants 2 and 3 played both games (Table 1). For both games the same standardized procedure was used: Eight hours prior to the game, a pill (e-Celsius performance capsule^®^, BodyCap, Hérouville-Saint-Clair, France) for Tc measurement was ingested. For each player, the connection between the monitor (e-Celsius performance moniteur^®^, BodyCap, Hérouville-Saint-Clair, France) and the pill was checked one hour before the game. Then, players were equipped with a heart rate monitor (Acentas heart rate monitoring belt^®^, Acentas GmbH, Hoergertshausen, Germany). The lightweight tag of the tracking system (XLOCATE^®^, Axiamo, Biel, Switzerland) was securely mounted on the wheelchair frame, as close as possible to the pivot and point of rotation. Additionally, players were asked to fill out a questionnaire about their nutrition, caffeine, alcohol and fluid intake, medication, amount of training (minutes) and sleep duration (hours) and quality (rating 1 to 10; arbitrary units) over the last 24 h. The amount of caffeine was assessed in the number of coffees, with the assumption that a cup of coffee contains 80 mg of caffeine. Additionally, it was exactly reported at which time the coffee was ingested. Fluid intake and alcohol were assessed by the players saying what and how much (in liters) they drank.

Athletes performed an individual warm-up with a duration of 15 min, Tc and heart rate were collected during the warm-up. Immediately after the warm-up, athletes were weighed (PUA579-CS300^®^, Mettler Toledo, Columbus, OH, USA) in their WCB chair (without the match jersey), and the drinking bottles were weighed (XS402S^®^, Mettler Toledo, Columbus, Ohio, United States) as well. Immediately before the game, participants were asked to rate their ThS on a visual analog scale ranging between −4 and +4 [24] and the perceived exertion on a Borg-scale from 6 to 20 [25]. Data collection of the performance parameters started with the beginning of the match clock and was only paused during a time-out, breaks between quarters and when a player was on the bench. During the game Tc was collected every 10 s. Heart rate was measured with a remote monitoring system with a sample rate of one Hz (Acentas Herzfrequenz Monitoring Team System^®^, Acentas GmbH, Hoergertshausen, Germany) and was paused analog to the tracking system. Playing time for each participant was assessed by a commercially available stopwatch (DELTA^®^, Sport-Thieme Germany, Grasleben, Germany). Water bottles were weighed throughout the game. Directly after the game, athletes and bottles were weighed again in their WCB chair (without the match jersey). Rating of ThS and perceived exertion were assessed, and Tc data were transferred from the pill to the monitor. Players were not allowed to use cooling techniques (i.e., water spray, cooling vest, cooling garments, ice packs, ice slurry) prior to or during the game.

### 2.3. Indoor Tracking System (ITS)

The ITS (XLOCATE^®^, Axiamo, Biel, Switzerland) is a position-tracking system based on ultra-wideband signals. Six wireless reference antennas were located around the playing field, one in each corner and two parallel to the halfway line to have optimal court coverage. The tags mounted on wheelchairs emit ultra-wideband signals and were tracked by means of time difference of arrival and triangulation. Tag signals were sampled at 20 Hz with a precision of ± 20 cm. The fully mobile configuration is based on wireless technologies and battery-powered devices. The tags come in a small form factor and are lightweight (size = 50 × 80 × 9 mm, weight = 30 g). Sensors were calibrated prior to the mounting on the wheelchairs by using the Axiamo software (Axiamo, Biel, Switzerland). Raw data files were exported and filtered using a 3-pass sliding-average filter with a window width proportional to the tag frequency [26]. Afterward, total distance (m) and mean velocity (Vmean, m.s-1) were calculated.

### 2.4. Fluid Balance

Fluid intake was carefully assessed by weighing drinking bottles before, during and after the game. Athletes dried themselves with a towel and were weighed (without the match jersey) in their wheelchairs. SR was calculated using Equation (1). The content of the drinking bottles was water.

(1) $$Sweat\;Rate\;\left(L/h\right)=\frac{pre\;body\;weightmass\;\left(Kg\right)\;–\;post\;exercise\;body\;weightmass\left(Kg\right)+fluid\;intake\left(Kg\right)}{exercise\;time\;\left(h\right)}$$

### 2.5. Equipment/Location

Games were played in an indoor sports complex with hardwood flooring, typical for indoor wheelchair team sports courts. The WCB court has the same dimensions as the court for AB basketball players. Environmental temperature and rh were measured with a commercially available thermo- and hygrometers (Irox JB913R^®^, OS Technology AG, Guemligen, Switzerland)

### 2.6. Statistics

Data were analyzed using the software R (R Foundation for Statistical Computing version 3.6.0; Vienna, Austria). Figures were created with the R-package “ggplot2”. After checking all measured parameters for normality using Shapiro Wilk’s tests, the median and interquartile range (IQR) were calculated for all parameters. Due to nonparametric data, mean differences between both games were explored using Wilcoxon’s rank-sum tests. Spearman’s coefficient was calculated to describe a correlation between parameters. Thereby, r < 0.3 shows none, 0.3 < r < 0.5 weak, 0.5 < r < 0.7 moderate, r > 0.7 strong correlation. The results were evaluated within a 95% confidence interval and at a statistical significance level of *p* < 0.05.

## 3. Results

### 3.1. Change in Body Mass

Within each game, body mass did not significantly change (HOT, *p* = 0.185; TMP, *p* = 0.125). The median change in body mass was significantly higher in the HOT (−0.35%, IQR: 0.15%) compared to the TMP (+0.11%, IQR: 0.35%, *p* = 0.015). Considering each participant individually, the data showed that four out of the five players lost body mass during the HOT, whereas in the TMP, all eight players gained body mass. More details are indicated in Table 2.

### 3.2. Fluid INTAKE

The median fluid intake per hour was 0.68 L in the HOT (IQR: 0.58 L) and 0.55 L in the TMP (IQR: 0.12) and was not significantly different between the games (*p* = 0.720) (Table 2).

### 3.3. Sweat Rate

The median SR for the HOT was 0.93 L/h (IQR: 0.58, range: 0.54–1.51 L/h) and 0.48 L/h (IQR: 0.19, range: 0.2–0.74 L/h) for the TMP and was significantly larger in the HOT (*p* = 0.020) (Table 2). Although SR was higher in the HOT compared to the TMP, and fluid intake was not significantly different between the two conditions (Table 2). The fluid intake rate per hour showed no difference compared with SR either in the HOT (*p* = 0.188) nor in the TMP (*p* = 0.125).

### 3.4. Body Core Temperature, Perceived Thermal Sensation, Game Characteristics and Pregame Behavior

**Figure 1:**
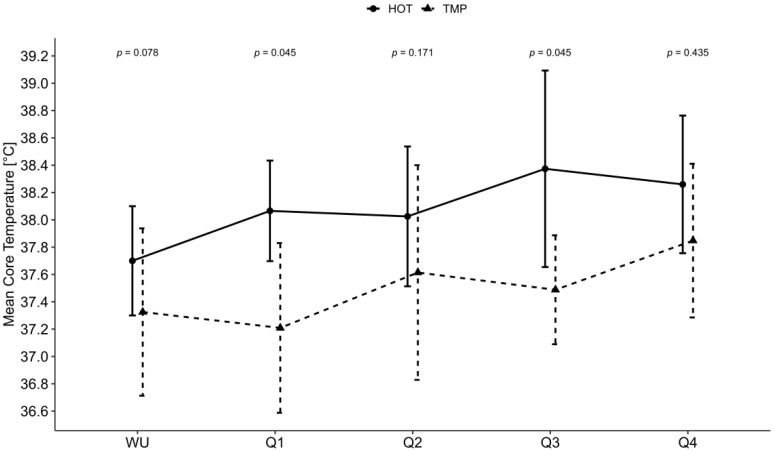
Median body core temperature (participants’ mean values per quarter) during warm-up and for each quarter. WU = warm-up; Q = quarter; HOT = game in hot conditions; TMP = game in temperate conditions; °C = degree Celsius; *p* = *p*-value; Error bars demonstrate inter quartile range and *p*-values present the meaningfulness of the difference between the two conditions. Significance level at *p* < 0.05.

**Figure 2:**
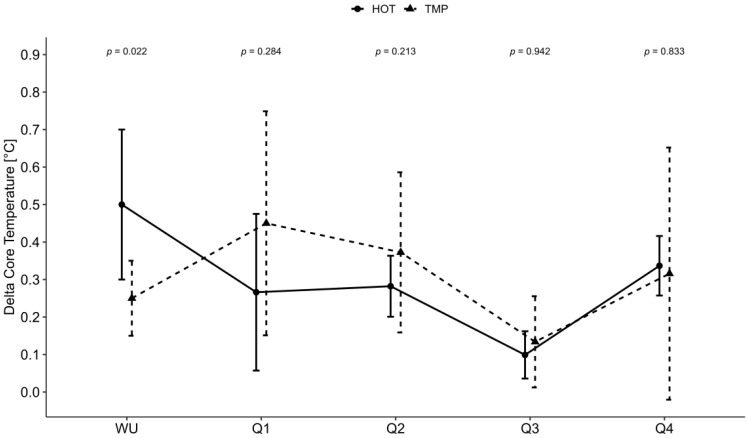
Increase in body core temperature during warm-up and during each quarter; WU = warm-up; Q = quarter; HOT = game in hot conditions; TMP = game in temperate conditions; °C = degree Celsius; *p* = *p*-value. Error bars demonstrate interquartile range and *p*-values present the meaningfulness of the difference between the two conditions. Significance level at *p* < 0.05.

The increase in Tc from the start to the end of the game was significant for the TMP (*p* = 0.008), whereas in the HOT, the change tended towards significance (*p* = 0.063). The baseline Tc was significantly higher in the HOT compared to the TMP (median: 37.9 °C, IQR: 0.4 °C vs. 37.5 °C, 0.65 °C, *p* = 0.04). The increase in the Tc was significantly higher in the TMP compared with the HOT (*p* = 0.010). The increase in Tc during warm-up was significantly larger in the HOT compared with the TMP (*p* = 0.022). The development of Tc and the change in Tc are presented in Figure 1 and Figure 2.

**Figure 3:**
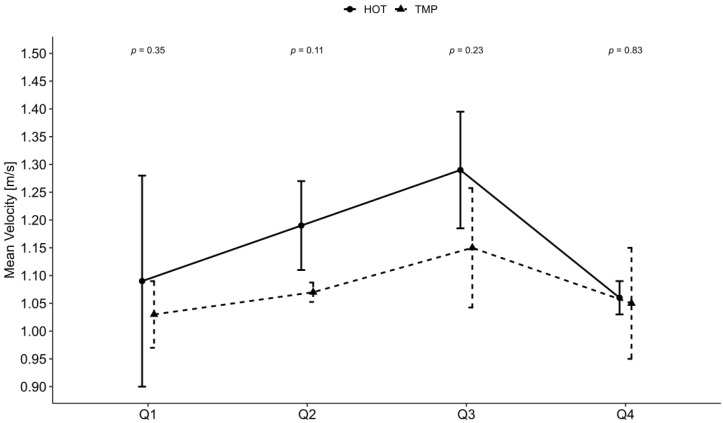
Mean velocity per quarter. Q = quarter; m/s = meter per second; *p* = *p*-value. Error bars demonstrate interquartile range and *p*-values present the meaningfulness of the difference between the two conditions. Significance level at *p* < 0.05.

At the end of the HOT, ThS was significantly higher (*p* = 0.008) compared with the beginning, whereas in the TMP no significant difference between the beginning and the end of the game was found (*p* = 0.063). All other measured parameters were not significantly different between both games (Table 2). The differences between the games concerning mean velocity (*p* = 0.540) and playing time were also not significant (*p* = 0.170). Figure 3 presents the mean velocity split up into quarters.

Descriptive statistics of caffeine intake in the last three hours before the games did not reveal a difference between the two games and was in median 80 mg for both games. The median fluid intake prior to the last two hours before the games was 0.5 L for both games. Nutritional intake was similar in both games (i.e., pasta with sugo). In both games, athletes reported a sleep quality between 7 and 10 with a duration between 7 and 8 h.

### 3.5. Correlations

The relative change in body mass was correlated with relative fluid intake in the HOT (r = 1, *p* = 0.017) but not in the TMP (r = 0.35, *p* = 0.389). A tendency for a correlation between SR and fluid intake rate was observed in the HOT (r = 0.90, *p* = 0.084) but not in the TMP (r = 0.62, *p* = 0.115). Fluid intake rate was not affected by the playing time (HOT, r = −0.30, *p* = 0.68; TMP, r = −0.67, *p* = 0.083), and a higher SR was not related with a lower increase in Tc (HOT, r = −0.15 *p* = 0.8; TMP, r = 0.46, *p* = 0.25). Additionally, the data did not show any correlation between the players’ classification (Table 1) and SR (HOT, r = 0.015, *p* = 0.932; TMP, r = 0.049, *p* = 0.912). In the HOT, there was a significant correlation between the change in ThS and Tc (r = 0.97, *p* = 0.005), as well as between the classification and relative body mass loss (r = 0.97, *p* = 0.004) and relative fluid intake (r = 0.97, *p* = 0.005).

### 3.6. Athletes with Paired Data

**Figure 4:**
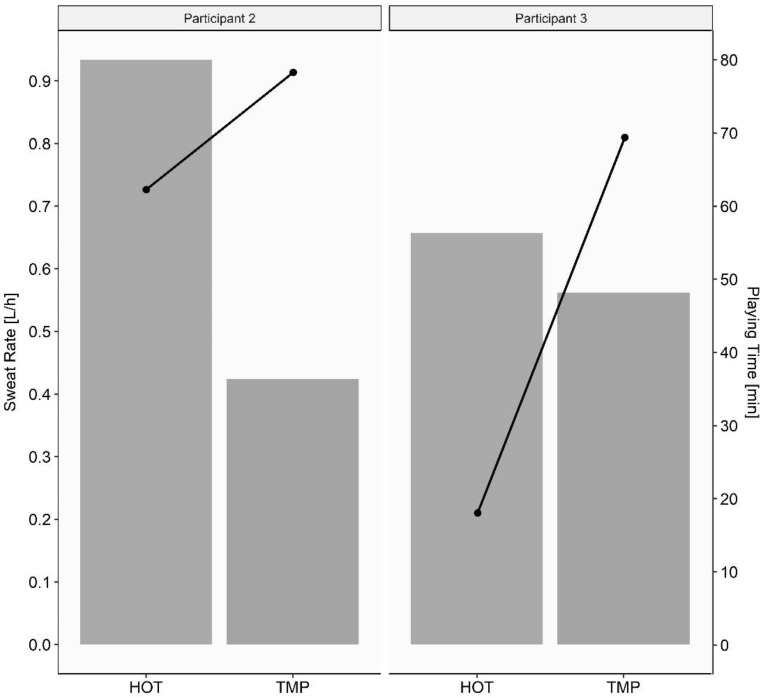
Sweat rate (bars) and playing time (black dots) of the two athletes who played both games. HOT = game in hot conditions; TMP = game in temperate conditions; L/h = liter per hour; min = minutes.

**Figure 5:**
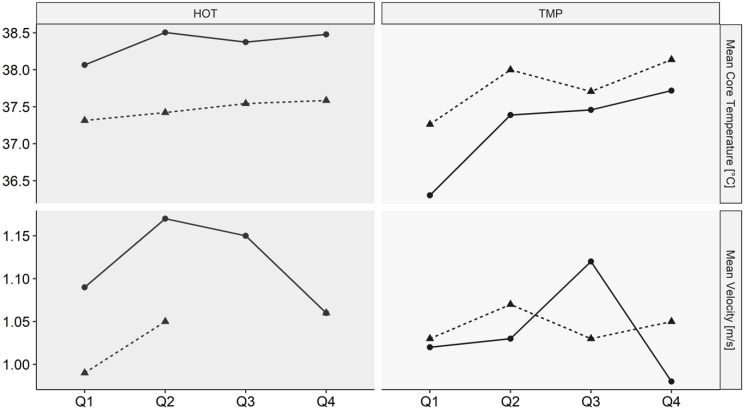
Mean body core temperature and mean velocity per quarter of the two players who played both games in the HOT and the TMP. Points represents participant 2, triangles participant 3; HOT = game in hot conditions; TMP = game in temperate conditions; Q = Quarter; °C = degree Celsius; m/s = meter per second.

Individual data for the two players who played both games are displayed in Figure 4 and Figure 5.

## 4. Discussion

This study investigated fluid balance and physiological responses of two WCB games, one in TMP the other in HOT conditions. The SR and body weight loss were significantly higher in the HOT compared with the TMP, whereas the increase in Tc was significantly higher in the TMP. Performance parameters did not differ between the two games.

### 4.1. Fluid Balance

Laboratory studies performed with AB participants have shown a lower cognitive, technical or endurance performance with a body mass loss of more than 2% [11,12]. The loss of fluid through sweating is widely variable and depends on different parameters (i.e., environmental conditions, clothing, level of impairment, exercise intensity and individual sweat capacity) [27]. The present investigation showed that the median of relative body mass loss (post-weight minus pre-weight) was 0.32% in the HOT, and in the TMP, the athletes even gained weight (+0.11%). Therefore, any decrease in physical or cognitive performance would not be expected. Furthermore, no difference in the mean velocity was observed between the HOT and the TMP (1.12 vs. 1.07 m/s). The SR was significantly higher in the HOT, but it seems that the athletes were able to compensate the higher fluid loss with a slightly larger fluid intake rate (0.68 L/h in HOT vs. 0.55 L/h in TMP). This is in line with other studies on the population of SCI. Logan-Sprenger and Mc Naughton [19] concluded that during official WCB games, athletes showed a good hydration strategy with a sufficient overall fluid intake (0.7 L) and with a minimal loss (0.4%) of body mass. During a 60 min arm crank exercise in temperate conditions, PA consumed ~ 0.4 L fluid and had a sweat loss of ~ 0.8 L [28]. In hot conditions, Price and Campbell [21] found a similar fluid consumption for PA (~0.4–0.5 L) but a higher intake in TP (0.75 L). The total sweat losses of 0.3 L for TP and ~0.7 L for PA [21] were slightly lower than those found in the present work, as well as those found by Price and Campbell [28] in the temperate conditions. During basketball games in AB, the SR was reported as 0.95 ± 0.42 L/h in temperate conditions (~23 °C) [29]. This was similar to our findings during the HOT (0.93 L/h), whereas in the TMP, athletes only reached about half (0.48 L/h) of the SR. This is another indication that athletes with SCI have a limited sweat capacity, since AB players sweat nearly the same amount in temperate conditions as SCI players in hot conditions. Body mass loss in our TMP condition was similar to AB basketball (~1%) [10]. This confirms previous findings that in most team sports, there are enough drinking opportunities to avoid hypohydration [12,30,31,32,33]. The fact that no correlation between fluid intake and playing time was found further confirms that enough game interruptions were present in WCB to use for fluid intake. Individual data showed that some players had a higher fluid intake than fluid loss, leading to a weight gain and fluid overconsumption. This shows that athletes with SCI have to be careful not to overhydrate in order to avoid an unnecessary weight gain that can influence performance [34]. Individual drinking protocols seem appropriate to consider factors such as individual sweat capacity or environmental conditions.

Comparing the two players who played both games, SR was higher (up to 0.5 L/h) for both in the HOT, despite a lower playing time than in the TMP (Figure 4). This demonstrates that WCB players with incomplete TP or a complete high-level PA still have a good thermoregulatory function and that they are able to increase their SR when heat stress is increasing. During exercise at a higher intensity, metabolic heat production would be expected to be higher. This could lead to more difficulties in the regulation of heat stress in those players [5].

The loss of electrolytes while sweating must not be neglected and has to be supplemented if necessary. In the present study, several athletes had a high SR, and since they were drinking only water, there might be a risk for hyponatremia. Thus, further research has to address the additional measurement of sodium and potassium concentration in the sweat to make individual advice for athletes’ fluid intake and potential electrolyte supplementation.

### 4.2. Body Core Temperature and Perceived Thermal Sensation

The current evidence [5] shows that the thermoregulation of individuals with a low-level SCI is not always negatively affected by their injury and they were able to regulate their Tc to the same level as observed in AB. This also applies when exercising in hot conditions [5]. Nevertheless, Tc has never been assessed during a competitive WCB in hot conditions. The presented results are in line with a previous laboratory study [21]. The increase in Tc was not larger in the HOT compared to the TMP. Interestingly, the increase during the HOT was even slightly lower (HOT, 0.8 °C; TMP, 1.05 °C). Some important considerations should be taken into account when interpreting these findings. Firstly, athletes had a higher baseline Tc in the HOT (37.9 °C) compared to the TMP (37.5 °C). One could argue that this reduces the heat storage capacity [35]. Furthermore, it could be that the higher baseline temperature as well as the higher environmental temperature led to an earlier onset of sweating [36]. This is partially supported by the higher SR in the HOT with a higher heat loss through evaporation. Secondly, the increase in Tc during warm-up was significantly higher in the HOT compared to the TMP. Since there was no intensity measurement of the warm-up, it can only be speculated that the warm-up was more intense in the HOT or by similar intensity the hot conditions must have further increased Tc. Thus, thermoregulatory responses might have been triggered at an earlier point in time, which reduced the increase in Tc during the game. Furthermore, the longer duration of the TMP (effective playing time was 4 x 10 min, but the overall duration was over 90 min) has led to a larger overall activation of the body and therefore a larger potential for Tc to increase. On the other side, the higher mean Tc in the TMP can possibly be attributed to the larger number of athletes with a higher level of lesion, which participated in the TMP. The subjective ThS did not differ between the games and was rated with a median of +2 in both games. This reflects a "moderate" sensation of perceived heat. This is another indicator that WCB athletes were able to regulate their Tc at a sufficient level. Since in both games the median ThS did not differ but the increase in Tc was significantly higher in the TMP and athletes reached higher values in the HOT, the ThS may not be a reliable tool for self-monitoring temperature during activity. Considering the two players who played both games, there is no noticeable difference in the dynamic of Tc, other than the higher baseline Tc in the HOT. Interestingly, in participant 3, the Tc rose continuously, even when the athlete did not play in the third quarter in the HOT (Figure 5). This shows that heat accumulation continued during rest, similar as Griggs et al. [37] had reported. Moreover, it can be argued that the hot environmental conditions lead to an imbalance between heat accumulation and dissipation.

### 4.3. Level of Lesion/Classification

The current evidence shows that only individuals with a high lesion PA or a TP have problems with thermoregulation during exercise in hot conditions [5]. In wheelchair sports, the classification system is often related to the level of lesion and the completeness of the transection of the spinal cord [23]. In the present investigation, for both games, the data did not show a tendency for a connection between classification and Tc. It is speculated that the external heat strain was not high enough to provoke differences or that the low intensity during the games did not trigger a sufficient heat production to provoke differences between the classification. In the HOT, there is a tendency for a possible connection between the classification and the SR, and the classification is related to relative body mass loss and fluid intake. In other words, the more motor functions a player has, the larger the SR and the body mass loss were, which they compensated with a larger fluid intake.

### 4.4. Limitations

The recruitment of an appropriate number of participants is difficult since athletes with SCI represent a small group in the general population. The small number of participants included in this study, heterogeneity of the participants, different environmental conditions and the combination of athletes from all classification levels complicated a comparison. Therefore, the interpretation of the results limited the experimental character of the study, and the data may serve as pilot data. Additionally, due to different transfers and retirements in the Swiss team between the two games, only two players participated in both games.

Since the athletes were weighted in their WCB chairs, the sweat which was stored in the upholstery was not assessed. Therefore, underestimation of sweat loss might have been the case. Furthermore, athletes were only weighed before and after the game. Therefore, sweat loss cannot be related to the playing time or intensity during the different quarters. Additionally, urine-specific gravity was not measured prior to the games. Thus, it is not possible to state if athletes started well hydrated into the game, and therefore, the SR could have been influenced by the hydration status.

Each WCB game has its own characteristics regarding the game characteristics (e.g., the team tactics, number of substitutions, the calls of the referees, numbers of interruptions) and exercise intensity. Comparing the physiological outcome parameters of different games is therefore difficult. A standardized testing procedure under "simulated" conditions (e.g., field test) is needed to draw any final conclusion on the influence of heat on SR and fluid intake, as well as on performance in hot conditions. This testing procedure might be used to test different fluid and nutritional interventions to optimize performance.

### 4.5. Practical Application

The presented data showed a significantly higher SR and relative body mass loss during a WCB game in hot conditions. Since the relative body mass loss was less than 2% and Tc was not significantly higher in the HOT, it is indicated that coaches and caregivers are already supporting the athletes with enough fluid, especially during the HOT, where the SR seems to increase. Nevertheless, during the HOT, special attention must be paid to this issue. On the other hand, athletes should be prevented from overhydration in temperate conditions. In more intense WCB games, the application of cooling strategies might be tested. Athletes have to be monitored for electrolyte concentration in sweat for optimal fluid intake strategy and supplementation of electrolytes.

## 5. Conclusions

In conclusion, this study observed a higher body mass loss and SR in a WCB game in the HOT compared to a game in TMP. Even though some athletes had a high SR, the body mass loss did not exceed the 2% barrier, which is associated with a performance decrement. Performance parameters and Tc were not negatively affected by the increase in environmental temperature. Thus, caregivers and athletes themselves were doing a good job in helping the athletes stay hydrated. Obviously, during both games, sufficient drinking opportunities were available to replace the lost fluid. Nevertheless, further research needs to collect data from official WCB games in HOT conditions with a larger number of participants to draw a more substantial conclusion. In addition, the measurement of individual sweat sodium and potassium concentration to optimize fluid intake strategies should be considered.

## Figures and Tables

**Table 1:** Participants’ characteristics.

Participant	Age [Years]	Lesion Level	Classification	Body Mass [kg]	Game Played	ITS
1	29.3	L1	4.0	82.8	HOT	x
2	39.7	C5	2.5	94.6	HOT, TMP	x
3	46.3	T5	1.0	78.6	HOT, TMP	x
4	40.2	T4	1.0	83.0	HOT	x
5	29.6	T10	3.0	70.0	HOT	x
6	42.4	L1	4.0	110.6	TMP	x
7	36.5	T12	2.5	65.4	TMP	x
8	59.7	T10	2.0	111.6	TMP	-
9	39.7	T10	1.0	70.6	TMP	-
10	54.0	L3	3.0	90.6	TMP	-
11	20.0	T8	1.0	74.2	TMP	-
Median, IQR	39.8, 11.3	-	-	82.8, 20.2	-	-

IQR = inter quartile range; L = lumbar; T = thoracic; C = cervical; kg = kilogram; HOT = game in hot conditions; TMP = game in temperate conditions; ITS = indoor tracking system; x = athlete was tracked; - = athlete was not tracked; Classification referred to [23].

**Table 2:** Thermoregulatory response during the two games.

	HOT		TMP		Difference
Measured Parameter	Median	IQR	Median	IQR	*p*
Change in Body Mass [%]	−0.35	0.15	+0.11	0.35	0.02 *
Fluid Intake [L]	1.08	0.91	1.08	0.25	0.94
Relative Fluid Intake [%]	1.01	1.18	1.25	0.42	0.83
Fluid Intake Rate [L/h]	0.68	0.58	0.55	0.12	0.72
Sweat Rate [L/h]	0.93	0.58	0.48	0.19	0.02 *
∆ Body Core Temperature [°C]	0.8	0.4	1.05	0.15	0.01 *
Max. Core Temperature [°C]	38.6	0.6	38.3	0.5	0.50
Mean Heart Rate [bpm]	122	22	119	31	1.00
∆ Thermal Sensation	2	1	2	1	0.45
Max. Thermal Sensation	3	0	3	1	0.11
Mean Velocity [m/s]	1.12	0.11	1.07	0.08	0.54
Playing Time [min]	54.8	27.0	70.3	38.6	0.17

HOT = game in hot conditions; TMP = game in temperate conditions; max = maximal; % = percent; L = liter; L/h = liter per hour; ∆ = change; °C = degree Celsius; bpm = beats per minute; m/s = meter per second; min = minutes; IQR = interquartile range; *p* = *p*-value; * = significant at a level of *p* < 0.05.

## Data Availability

The datasets used and analyzed during the current study are available from the corresponding author on reasonable request.
